# ‘Side effects’ are ‘central effects’ that challenge retention in HIV treatment programmes in six sub-Saharan African countries: a multicountry qualitative study

**DOI:** 10.1136/sextrans-2016-052971

**Published:** 2017-07-23

**Authors:** Jenny Renju, Mosa Moshabela, Estelle McLean, William Ddaaki, Morten Skovdal, Fred Odongo, Dominic Bukenya, Joyce Wamoyi, Oliver Bonnington, Janet Seeley, Basia Zaba, Alison Wringe

**Affiliations:** 1 Faculty of Epidemiology and Population Health, London School of Hygiene and Tropical Medicine, London, UK; 2 Malawi Epidemiology and Intervention Research Unit, Karonga, Malawi; 3 Africa Health Research Institute, KwaZulu Natal, South Africa; 4 University of KwaZulu Natal, KwaZulu Natal, South Africa; 5 Rakai Health Sciences Program, Kalisizo, Uganda; 6 Department of Public Health, University of Copenhagen, Copenhagen, Denmark; 7 Biomedical Research and Training Institute, Harare, Zimbabwe; 8 Kenya Medical Research Institute, Nairobi, Kenya; 9 Medical Research Council/Uganda Virus Research Institute Research Unit on AIDS, Entebbe, Uganda; 10 National Institute for Medical Research, Mwanza, Tanzania

**Keywords:** AFRICA, AIDS, QUALITATIVE RESEARCH, HIV

## Abstract

**Objectives:**

To explore the bodily and relational experience of taking antiretroviral therapy (ART) and the subsequent effect on retention in HIV care in six sub-Saharan African countries.

**Methods:**

In-depth interviews were conducted with 130 people living with HIV (PLHIV) who had initiated ART, 38 PLHIV who were lost to follow-up and 53 healthcare workers (HCWs) in Kenya, Uganda, Tanzania, Malawi, Zimbabwe and South Africa. PLHIV were purposely selected to include a range of HIV treatment histories. Deductive and inductive analysis was guided by aspects of practice theory; retention in HIV care following ART initiation was the practice of interest.

**Results:**

PLHIV who were engaged in HIV care took ART every day, attended clinic appointments and ate as well as possible. For PLHIV, biomedical markers acted as reassurance for their positive treatment progression. However, many described ART side effects ranging from dizziness to conditions severe enough to prevent them from leaving home or caring for themselves or others. In all settings, the primary concern of HCW was ensuring patients achieved viral suppression, with management of side effects seen as a lower priority. Where PLHIV tolerated side effects, they were deemed the lesser of two evils compared with their pre-ART illnesses. Participants who reported feeling well prior to starting ART were often less able to tolerate side effects, and in many cases these events triggered their disengagement from HIV care.

**Conclusions:**

Retention in ART care is rarely an outcome of rational decision-making, but the consequence of bodily and relational experiences. Initiatives to improve retention should consider how bodily experiences of PLHIV relate to the rest of their lives and how this can be respected and supported by service providers to subsequently improve retention in care.

## Background

Over the past decade, significant achievements have been made in bringing HIV care and treatment to people living with HIV (PLHIV) in sub-Saharan Africa, with approximately 13 million people on antiretroviral therapy (ART) by the end of 2013.^1^ The transformational effects of ART are unquestionable, and the successful scale-up of national HIV treatment programmes has enabled ambitious goals to be established to eliminate AIDS by 2030. This target can only be reached by achieving high rates of testing, initiation of ART and viral suppression among PLHIV.^1^ ^2^ However, systematic reviews of retention in HIV care show suboptimal outcomes in many African settings which can jeopardise individual health outcomes and may undermine public health initiatives to eliminate AIDS.^3^ ^4^

ART side effects are a recognised factor that can undermine treatment success.^5^ From a clinical and public health perspective, the benefits of viral suppression that result from adherence to ART outweigh the risks associated with side effects or uncontrolled viral replication. The extent to which PLHIV perceive the trade-off in the same terms and over time is less clear. Side effects have been reported with the use of all ART drugs,^6^ with a recent study in three African countries finding that 88% of participants described at least one side effect during the preceding four weeks. Fatigue, loss of energy, pain, numbness, headache and depression were among the most frequently cited side effects.^7^ The same study found that every additional self-reported side effect increased the odds of incomplete adherence by 10%.^8^ Few qualitative studies have explored how these experiences affect the daily lives of PLHIV and subsequently contribute to sustained or suspended retention on ART. In this paper, we explore the bodily and relational experience of taking ART and the subsequent effect on retention in HIV care in six sub-Saharan African countries.

### Theoretical framework

Retention is an ongoing process corresponding to a re-enactment of various practices over time in different social contexts. For PLHIV, these include regularly attending HIV clinics, taking ART on a daily basis, adhering to advice on ‘healthy living’ provided by healthcare workers (HCWs) and adopting strategies to mediate the side effects of treatment. Drawing on aspects of practice theory^9^ and the concept of ‘situated rationalities’,^10^ we explore how the perceived and actual ability or willingness of PLHIV to engage with the clinical requirements of HIV treatment are influenced by the experiences of their everyday activities, such as work or social relationships, and how these aspects of their lives relate to each. Practice theory unpicks the dynamic relationship between social structure and human agency. Through a situational analysis we will be able to disentangle how diverse social beings (in terms of motives and intentions) form and are formed by the ‘rationalities’ of the world which they live in.

## Methods

We draw on data from a large qualitative study investigating health-seeking experiences of PLHIV in seven health and demographic surveillance sites (HDSS) in six African countries: Uganda (Kyamulibwa, Rakai), Tanzania (Kisesa), South Africa (uMkhanyakude), Kenya (Kisumu), Zimbabwe (Manicaland) and Malawi (Karonga).^11^

Sampling frames were constructed for the larger study using HDSS datasets and HIV clinic records to purposively sample PLHIV with a range of care and treatment histories (n=264). A purposive selection of HCWs with experience in providing HIV diagnosis, care or treatment services (n=53) and family members of PLHIV who had died (n=48). Additional methodological details concerning the Bottlenecks study can be found in the online supplementary material found within the editorial at http://dx.doi.org/10.1136/sextrans-2017-053172.^12^ For the purpose of this analysis, only PLHIV who had already initiated ART (n=168) and HCWs (n=53) were included (table 1) (see online supplementary file).

**Table 1:** The study participants shown by sampling category and country

Country	Demographic surveillance site	HCWs	PLHIV	
			On ART*	LTFU†
Uganda	Rakai	6	15	6
Uganda	Kyambuliwa	5	8	4
Kenya	Kisumu	8	15	6
Tanzania	Kisesa	7	20	4
Malawi	Karonga	5	20	4
Zimbabwe	Manicaland	4	35	8
South Africa	uMkhanyakude	18	17	6
	Total	53	130	38

The number of PLHIV sampled per setting varied due to the secondary sampling objective for the wider study to include other variables (rather than just care and treatment trajectories) that may influence the experiences of PLHIV in accessing HIV services in their setting, such as their pregnancy status, area of residence or livelihood, etc. The number of HCWs varied in accordance with the number and types of clinics and HCWs in the HDSS providing HIV care.

*The PLHIV could have been taking ART for variable periods of time, countries had varying cut-off points but generally captured recently initiated and then longer term (over 5 years).

†PLHIV had not collected ART from their registered clinic for a country specific predetermined period of time.

ART, antiretroviral therapy; HCWs, healthcare workers; HDSS, health and demographic surveillance sites; LTFU, lost to follow-up; PLHIV, people living with HIV.

Face-to-face in-depth interviews (IDIs) lasted between 45 and 90 min and were conducted in private, either at homes or clinics, by trained and qualified interviewers who had previous experience of working in the HDSS. One-off IDIs with PLHIV captured their life circumstances and their experiences of HIV services and ART. One-off IDIs with HCW explored their rapport with PLHIV, experiences of delivering HIV services and their perceptions of the underlying drivers of retention on ART among PLHIV. Repeat interviews were conducted where necessary and ranged between 0 and 5 per site.

All IDIs were audio-recorded. In Kyamulibwa (Uganda), the recordings were used to develop detailed accounts including illustrative quotations, which were then translated into English. Elsewhere, the recordings were transcribed and translated into English. A broad coding framework was applied by each local site coordinator to organise the data in relation to care and treatment histories and other influences in the lives of PLHIV, with summaries produced to document emerging themes. The lead author reviewed these summaries and developed an analytical coding scheme based on elements of practice theory to explore the PLHIV experiences of taking ART and how this affected and was affected by the ‘rationalities’ of their daily lives. An inductive coding approach was adopted to allow a more nuanced exploration of emerging ideas (eg, experiences of side effects) and thematic areas (eg, relational experiences between previous experience of AIDS-defining illnesses and ability to cope with side effects).^13^ ^14^

## Results

The sample included 130 PLHIV who had initiated ART, 38 PLHIV who were lost to follow-up (LTFU) and 53 HCWs (table 1).

### Strategies and situations which helped to normalise taking ART and retention in care

PLHIV who were retained on ART reported taking their medication daily, attending clinic appointments and taking care to eat as well as possible. Many took ART after their evening meal and immediately prior to going to bed to mitigate frequently reported feelings of dizziness or weakness. Those who took their medication daily often described themselves as being well, with many aware of their CD4 counts or at the very least the utility of the measure, and some choosing to use this as a proxy measure of their health status.

Food insecurity remained an influential factor affecting retention on ART, with many reporting challenges in securing adequate food to maintain them while on drugs. Those who were unable to eat sufficient amounts reported that the dizziness intensified, forcing them to take the drugs just prior to sleeping. For many, this dizziness persisted even in the morning, with one woman explaining how in the morning she could still ‘smell’ the drugs if she had not been able to eat.

… when I haven't eaten something in the morning around 09:00 after taking the medicine [ART], my whole body stinks due to strength of the medicine … I need to eat food. (Female, PLHIV—on ART >5 years, Malawi)

### Experiences of side effects and engagement with HIV services

Many PLHIV, particularly those routinely taking their ART, reported favourably on their interactions with HCW and HIV services. The nature of the interactions was particularly pertinent when PLHIV were struggling to adjust to new treatments. When an explanation for their symptoms was offered, and support or encouragement given, it was often possible for PLHIV to continue taking ART.

I took these pills and they made me feel dizzy. I could not walk and would crawl to bed and I would see the house going in circles but I came back here they encouraged me to continue taking the pills.

(Female, PLHIV—on ART <5 years; Zimbabwe)

Acceptance, adjustment and management of life on ART were particularly problematic when side effects developed later, after already tolerating the adjustment period when first starting ART. Participants described debilitating side effects ranging from dizziness and headaches through to pains and conditions severe enough to prevent them from leaving the house or caring for themselves or their families. The consequences among PLHIV varied, with some unable to attend clinic and sending treatment partners to collect medication on their behalf, others reporting their challenges to HCWs and some interrupting treatment.

I started feeling dizzy. I felt it for some time but I just kept it to myself. I told nobody … I realised one tablet is the one which made me have that feeling and I stopped taking it. (Male, PLHIV–LTFU, Kenya)

### The tension between the need for viral suppression and the realities of dealing with side effects

HCWs in Kenya explained how, according to guidelines, they are supposed to counsel all PLHIV who need to initiate treatment three times prior to commencement:

Like when a client starts ART there are some side effects that he might see, we need to treat the client of the side effects, things like headache, drowsiness, loss of appetite, bad dreams, things like that of which we have to inform or alert the client, if he sees, these things will go away after 2–3 weeks, but the client is not supposed to stop the drugs, he is supposed to continue with the drugs, even things like rashes, they are so many, vomiting, so according to the guideline, before we initiate clients, we have to do first adherence, we do the second adherence after 2 weeks, then after one week we do the 3rd adherence, then we fill the pre HAART form for the client … we have to inform the client of the side effects appropriately, yeah and also to encourage the client not to stop the drugs, yeah. (HCW, Kenya)

However, in reality in all countries HCWs were reported to ‘ignore’ side effects or describe them as ‘normal’ while the PLHIVs' ‘bodies were adjusting to the drugs’ they were not used to (HCW, Kenya). While some HCWs recognised that PLHIV ‘stop taking medicine because of reactions’ (HCW, Malawi), in all settings, the HCWs’ primary focus was ensuring that PLHIV achieved viral suppression. HCWs prioritised adherence monitoring which necessitated stringent pill counts, and where available, viral load testing either with or without health education. The possibility of modifying treatment regimens to reduce side effects was rarely mentioned by either HCWs or PLHIV. The focus on viral suppression led to a lesser regard being placed on the experiences and challenges of coping with side effects. One PLHIV in Zimbabwe described that despite his efforts to articulate his daily struggles, the HCW was primarily focused on his need to initiate ART; this divergence of priorities ultimately led to him to disengage with care.

I had not taken them [ART] for many months because when I started taking them my face, hands and legs started swelling. Then they started peeling and my face becomes black … I went to see a doctor at Hauna, the doctor did not tell me anything that was satisfying, he just started by asking me what was happening to my face, I was burnt and dark. Then I told him it happened after I had taken the ARVs, my hands had peeled and my feet … I visited the doctor twice and they would say I should continue to take my medication. So I just wondered if I was safe because of the continuous reaction, so I stayed at my home and I stopped taking the medication. (Female, PLHIV–LTFU, Zimbabwe)

The interviews with the HCWs did elucidate a level of sympathy and understanding of the challenges that PLHIV faced. However, their actions were hindered by the constraints of their working environments including requirements to follow policies dictating when ART should be started, what drugs were available to them and how often to dispense.

### The relation between the benefits of ART and other aspects of life and treatment

**Figure 1:**
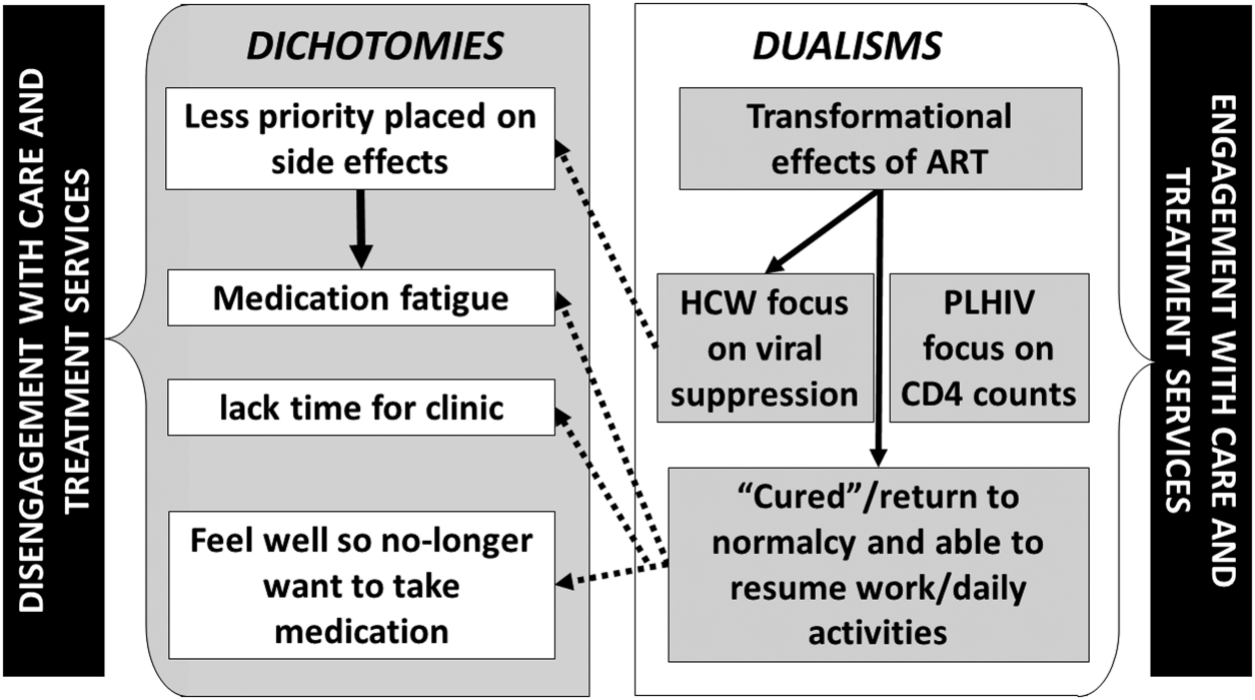
The relation between antiretroviral therapy and other aspects of life and treatment. ART, antiretroviral therapy; HCW, healthcare workers; PLHIV, people living with HIV.

The practices we have described above relate to other aspects of the PLHIV's lives that influence their ‘practice of retention’, thereby forming a mutually constituted duality or dichotomy (a concept embedded within theories of practice),^15^ which could either promote or inhibit engagement in care (figure 1).

Many PLHIV had experienced the transformational effects of ART and described their return to a ‘normal’ life. This generally referred to a restoration of activities associated with their lives before they were affected by HIV. It also related to how they perceived they should live in the context of social norms and expectations, for example, the imperative to stay alive to care for their dependents.

… I recall the way I was before I started ART and now, there's a big change so if I can just stop taking them now that means I will fall sick again that is the reason I don't stop. (Female, PLHIV—on ART—recently initiated, Malawi)

However, experiences of side effects muddied the continuum from illness to medication to ‘cure/normality’. In some instances, the side effects were tolerated as they were deemed the lesser of two evils in comparison to the illness that was experienced pre ART. However, others, particularly those who identified themselves as well prior to the prescription of ART, were less able to tolerate such debilitating side effects and thereby live ‘normal’ lives.

The requirement for viral suppression leading to the focus on adherence and physical pill counts, coupled with the hierarchy of power loaded on the side of the HCW, was a driver to adhere/comply. However, these strategies to promote adherence have dichotomies (as illustrated in figure 1), namely a lack of focus on the side effects which led PLHIV to stop taking ART.

The transformational effect of ART enabled many PLHIV to feel ‘normal’ again; some even reported to be ‘cured’. In this respect, they were able to resume their daily activities including returning to formal employment or farming, looking after the home and children. However, such activities made it harder for many participants (particularly those who had to travel for work) to attend their routine clinic visits. Further, there remains a strong incongruence between the feeling of being ‘cured’ or ‘normal’ and still having to take medication (see figure 1). We heard from one PLHIV who was LTFU that he ‘stopped because [he] was feeling well and was not feeling any pain in [his] body’ (female, PLHIV–LTFU, Zimbabwe). This was reinforced by many HCWs, one of whom said PLHIV ‘stop taking medicine because they feel they are healed’ (HCW, Malawi). Feeling ‘normal’ or ‘cured’ led others to not identify as sick, and therefore resist or disregard engagement with support groups which have been well established as important facilitators in retention in care.^16^

## Discussion

Retention in care is critical for the successful management of HIV and required in order to reach the United National Programme on HIV/AIDS ‘90-90-90’ targets.^1^ ^2^ To our knowledge, this is the first multicountry qualitative study to explore how the bodily and relational experiences of taking ART in different settings impact on retention on ART. Recent quantitative studies in similar settings found a relationship between the occurrence of side effects and incomplete adherence.^7^ Our in-depth qualitative study elucidates possible reasons for these increased levels of disengagement. In this paper, we illustrate that the ‘side effects’ of ART are actually ‘central effects’. Our findings suggest that side effects, either when adjusting to treatment or when they return or change, are not treated as serious complications, with a primary focus driven by a need to achieve viral suppression.

Our application of practice theory illustrates that relationality and dichotomies cannot be underestimated, with the presence of a constant tug between the biomedical requirements for clinic visits and treatment adherence to obtain viral suppression, on the one hand, and the immediate needs and desires of PLHIV to ‘get back to normal’, on the other.^16^ Our findings have implications as we move to the era of ‘test and treat’,^17^ where diagnosed PLHIV may not have experienced feelings of ill-health nor witnessed the transformational effects of ART. What will be the motivation for tolerating side effects that undermine ‘normality’? If the relationality is no longer strong enough, will people really make an effort to ensure that daily ART become an integral part of their lives? Our research would suggest that past illnesses are more likely to lead to greater tolerance of side effects and therefore earlier treatment prior to sickness or near death removes the point of comparison and could potentially limit the motivations required to tolerate them.

In all settings, PLHIV placed value on biomedical indicators such as CD4 counts as a proxy guide for their health status. A recent study in East Africa found that the lack of on-site CD4 testing negatively affected engagement in HIV care.^18^ Our findings suggest that the need to measure one's ‘biological health improvements’ will be affected if the role of CD4 counts becomes outdated, in light of the imminent ‘test and treat’ policies. The current attention on the need for accessible and affordable routine viral load testing to accurately monitor viral suppression^19^ will be crucial to support this primarily positive practice to measure one's health improvements.

The study demonstrates a continued dichotomy between the pull of the HCWs to comply with public health policies dictating their prescription of ART against the individual needs of each PLHIV. The experiences of PLHIV across the six countries support the need for differentiated care. Adopting a ‘one-size-fits-all’ practice pertaining to which ARTs, when and how, could carry negative and unintended consequences as individual PLHIV miss opportunities for more comprehensive assessments of their clinical and social needs.^19^

There are several strengths and limitations to our study. The HDSS databases and experienced research teams meant it was easier to locate, recruit and interview PLHIV who were no longer in HIV care, although in small numbers. However, as with many qualitative studies the small sample sizes (particularly when disaggregated by setting) limit intercountry comparisons. In each setting, data saturation was achieved, and during analysis, focus was placed on considering whether salient themes were more apparent in a given setting over another; however, in this instance, many of the experiences resonated across all the counties lending support to the wider generalisability of our results. Finally, our study was susceptible to social desirability bias, but this was minimised by ensuring that participants' accounts would remain confidential and training interviewers to avoid judgemental reactions.

## Conclusions

Our multicountry qualitative study highlights that retention is not an outcome of rational knowledge of what one should and should not do to be retained in care, but is largely an outcome of bodily knowledge and relational experiences. Such experiences are located in everyday routines and past and present bodily experiences. Initiatives to improve retention should consider how the bodily and relational experiences of PLHIV taking ART can be respected and supported by service providers to subsequently improve retention in care.

**Key messages**

- Many people living with HIV (PLHIV) placed value on biomedical indicators (eg, CD4 counts or viral loads) as a proxy guide for their health status. Availability of such indicators is crucial to positively support PLHIV to be retained in care.
- Side effects of antiretroviral therapy are not treated as serious complications by health workers who tend to be driven by a need to achieve viral suppression.
- Initiatives to improve retention should consider the potential consequences of side effects and how these are socially and relationally conditioned.
